# Geographic and Ecological Dimensions of Host Plant-Associated Genetic Differentiation and Speciation in the *Rhagoletis cingulata* (Diptera: Tephritidae) Sibling Species Group

**DOI:** 10.3390/insects10090275

**Published:** 2019-08-29

**Authors:** Meredith M. Doellman, Hannes Schuler, Gilbert Jean Saint, Glen R. Hood, Scott P. Egan, Thomas H.Q. Powell, Mary M. Glover, Daniel J. Bruzzese, James J. Smith, Wee L. Yee, Robert B. Goughnour, Juan Rull, Martin Aluja, Jeffrey L. Feder

**Affiliations:** 1 Department of Biological Sciences, Galvin Life Sciences Bldg., University of Notre Dame, Notre Dame, IN 46556, USA; 2 Advanced Diagnostics and Therapeutics, University of Notre Dame, Notre Dame, IN 46556, USA; 3 Michigan State University, Department of Entomology and Lyman Briggs College, East Holmes Hall, E. Lansing, MI 48824, USA; 4 United States Department of Agriculture, Agricultural Research Service, Temperate Tree Fruit and Vegetable Research Unit, 5230 Konnowac Pass Road, Wapato, WA 98951, USA; 5 Washington State University Extension, 1919 NE 78th Street, Vancouver, WA 98665, USA; 6 PROIMI Biotecnología-CONICET, LIEMEN-División Control Biológico de Plagas, Av. Belgrano y Pje. Caseros, T4001MVB San Miguel de Tucumán, Tucumán, Argentina; 7 Instituto de Ecología, A.C., Carretera Antigua a Coatepec no. 351, Congregación el Haya, C.P. 91070 Xalapa, Veracruz, Mexico; 8 Environmental Change Initiative, University of Notre Dame, Notre Dame, IN 46556, USA

**Keywords:** adaptive radiation, speciation, sympatry, allopatry, reproductive isolation

## Abstract

Ascertaining the causes of adaptive radiation is central to understanding how new species arise and come to vary with their resources. The ecological theory posits adaptive radiation via divergent natural selection associated with novel resource use; an alternative suggests character displacement following speciation in allopatry and then secondary contact of reproductively isolated but ecologically similar species. Discriminating between hypotheses, therefore, requires the establishment of a key role for ecological diversification in initiating speciation versus a secondary role in facilitating co-existence. Here, we characterize patterns of genetic variation and postzygotic reproductive isolation for tephritid fruit flies in the *Rhagoletis cingulata* sibling species group to assess the significance of ecology, geography, and non-adaptive processes for their divergence. Our results support the ecological theory: no evidence for intrinsic postzygotic reproductive isolation was found between two populations of allopatric species, while nuclear-encoded microsatellites implied strong ecologically based reproductive isolation among sympatric species infesting different host plants. Analysis of mitochondrial DNA suggested, however, that cytoplasmic-related reproductive isolation may also exist between two geographically isolated populations within *R cingulata*. Thus, ecology associated with sympatric host shifts and cytoplasmic effects possibly associated with an endosymbiont may be the key initial drivers of the radiation of the *R. cingulata* group.

## 1. Introduction

Adaptive radiation has been defined as “the evolution of ecological diversity within a rapidly multiplying lineage” [1]. Adaptive radiation is important because it marks the interface of ecology and evolution and is perhaps the most common mechanism for the origin and proliferation of species [1,2]. Thus, determining the causes of adaptive radiation is central to understanding how new life forms arise and come to vary with the resources they use and environments they inhabit—foundational issues in evolutionary biology.

The ecological theory posits that adaptive radiation is due primarily to divergent natural selection stemming from variation in environmental factors, such as resource availability and competition, that drive phenotypic divergence, population differentiation, and ultimately, speciation [1]. A key component of the theory is ecological opportunity: the existence of open niches that can be exploited allowing for ecological diversification [3,4]. New or open niches can become available when: 1) a species colonizes a new area lacking competitors, 2) the competitors of a species go extinct, 3) a species evolves a key innovation that allows it to interact with the environment in a novel way, and/or 4) a species constructs new niches that transcend trophic levels, creating resources for other members of a community to utilize and diversify in kind [1,4,5]. Thus, the ecological theory predicts a chronology in which divergent natural selection and the subsequent adaptation of populations to use new resources and/or to avoid competitors, parasites, or predators, repeatedly and rapidly occurs to generate reproductive isolation (RI) that initiates speciation and ultimately, creates a diverse clade of taxa.

There are alternatives to the ecological theory, however. For example, adaptive differentiation could also occur following non-ecological speciation [6]. In this case, “allospecies” form first, as geographically isolated populations independently accumulate genetic differences due to directional selection and/or drift that result in the evolution of intrinsic postzygotic RI, such as Batson–Dobzhansky–Muller genomic incompatibilities [7,8]. Subsequently, upon secondary contact, ecological character displacement may occur in which one or both of the taxa phenotypically diverge to avoid being competitively excluded and going extinct [9,10]. If such a process occurs repeatedly and quickly, then a pattern of adaptive radiation would result.

Understanding the basis for adaptive radiation, therefore, requires one to distinguish whether ecological diversification has played a primary role in initiating speciation versus a secondary role in allowing taxa to co-exist and persist after speciation. Both scenarios are viable and there are examples in the literature supporting the ecological and non-ecological hypotheses (reviewed in [1,6]). From a compilation of such studies, generalities may eventually emerge about the relative significance of ecological versus non-ecological processes in adaptive radiation and the origin of species.

Empirically resolving ecological and non-ecological explanations for initial divergence during adaptive radiation, however, can be difficult [1,6,11]. First, this requires confirmation of a system of closely related and rapidly diversifying species undergoing adaptive radiation. Second, details must be known concerning the phylogenetic relationships, biogeography, natural history, and biology of the study system, including the basis and origins(s) of RI, to distinguish between the ecological versus non-ecological hypotheses. Finally, we portrayed adaptive radiation above as initiated by either ecological or non-ecological speciation. However, both processes may contribute to and interact during adaptive radiation [2]. For example, an evolutionary innovation that opens new niche opportunities may initially facilitate ecological speciation. Newly derived taxa freed from competition, predation, parasitism and/or abiotic constraints, may subsequently expand their ranges into areas where they were previously excluded, potentiating the opportunity for non-ecological, allopatric speciation. If these taxa come back into secondary contact, then character displacement could occur, further promoting ecological diversification in the clade. Moreover, character displacement could itself help spawn new species if the resulting ecological divergence in the area of overlap generates RI with formerly conspecific populations in different regions. Finally, even in situations where allopatrically separated populations are associated with different habitats, both adaptive and non-adaptive processes may contribute to the evolution of RI and speciation.

Given these considerations, it would seem that an ideal system for resolving processes from patterns in adaptive radiation would be to study a group of related taxa that are at varying stages of speciation from one another and resolve the degree to which they are isolated by ecological versus non-ecological barriers to gene flow [12]. Although such an approach does not document the divergence process from start to finish for any one pair of taxa, reasonable inferences may still be made concerning whether divergent selection during speciation or character displacement after speciation formed the basis for ecological diversification. Moreover, the historical insight provided by such an approach can also reveal whether and how ecological and non-ecological processes interact to generate adaptive radiation.

Tephritid fruit flies in the genus *Rhagoletis* are models for adaptive radiation in phytophagous insects [13,14,15,16,17]. In North America, the genus contains species groups, such as the *R. pomonella* and *R. tabellaria* sibling species groups, that have radiated sympatrically via shifting to and ecologically specializing on new host plants [18,19,20]. The most well-known example is the recent shift of *R. pomonella* from its native host plant hawthorn (*Crataegus* spp.) to the introduced, domesticated apple (*Malus domestica*) in the mid-1800s in Eastern North America [14,18,21]. However, other *Rhagoletis* species groups, such as the *R. suavis* group of walnut husk flies, all attack the same genus of host plants (*Juglans* for *R. suavis*) [18,19,22,23]. Unlike *R. pomonella* and *R. tabellaria* group flies, all the members of the *R. suavis* group are allopatric or parapatric and are morphologically distinct, with several species displaying sexual dimorphism in body coloration. It has, therefore, been proposed that walnut husk flies primarily diverged by an allopatric mode, through a combination of sexual selection and the evolution of intrinsic postzygotic RI [18,22,23].

**Figure 1:**
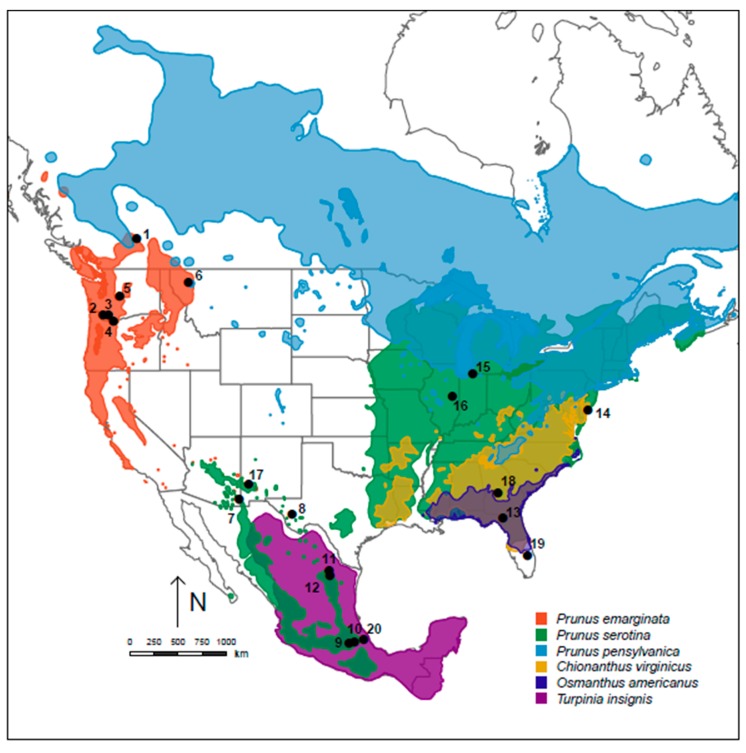
Map of collecting sites. Shown are numbered sampling locations for *R. cingulata* group flies from across North America. See Table 1 for designations and descriptions of study sites. Green indicates the range of *Prunus serotina*, black cherry; orange the range of *P. emarginata*, bitter cherry; dark blue the range of *Osmanthus americanus*, the tea olive or devilwood; gold the range of *Chionanthus virginicus*, the fringe-tree [28,29,30]. The range of *Turpinia insignis* (purple), muttonwood, was estimated from [31]; however, see Figure S1 for the range of *T. insignis* in Veracruz, Mexico, the only known site of infestation with *R. turpiniae* [32,33,34,35]. Also shown in blue is the range of *P. pensylvanica*, the pin cherry, that was not sampled in the current study but could potentially be a host bridging the Eastern USA population of *R. cingulata* with the Northwestern USA and Canadian population of *R. indifferens*.

Members of the *R. cingulata* sibling species group, the focus of the current study, share features with the *R. pomonella*, *R. tabellaria*, and *R. suavis* groups [18]. Like the sympatrically diverging *R. pomonella* and *R. tabellaria* group flies, members of the *R. cingulata* group are frugivorous specialists on a variety of different host plants, including cherries, black cherry *Prunus serotina* Ehrh (principal host of *R. cingulata* Loew, eastern cherry fruit fly) and bitter cherry *P. emarginata* (principal host of *R. indifferens* Curran, western cherry fruit fly); native olives, tea-olive or devilwood *Osmanthus americanus* Sligh (host of *R. osmanthi* Bush) and fringe-tree *Chionanthus virginicus* W.S. Earle (host of *R. chionanthi* Bush); and muttonwood *Turpinia insignis* Kunth (host of *R. turpiniae* Hernandez-Ortiz). Similar to *R. pomonella*, the olive-infesting flies are sympatric with the more widely distributed *R. cingulata* in the Southeastern USA, while *R. turpiniae* co-occurs with *R. cingulata* in Southern Mexico (Figure 1). Finally, while cherry-infesting flies display a polymorphism for an apical wing spot, some other slight differences in leg coloration and, in males only, a single spot on the dorsal side of one abdominal segment, in general, *R. cingulata* group species are morphologically similar to one another [18].

*Rhagoletis cingulata* group flies also share attributes with the allopatrically speciating *R. suavis* group. For example, *R. cingulata* and *R. indifferens* do not overlap geographically in their ranges in North America (Figure 1). Moreover, while the primary native hosts of *R. cingulata* and *R. indifferens* differ (black cherry versus bitter cherry, respectively), both flies attack other cherries in common, including introduced domesticated sweet cherries (*P. avium* L.) and tart cherries (*P. cerasus* L.), as well as the native pin cherry, *P. pensylvanica* L.f. [18]. Thus, cherry host affiliation may not be a strong barrier to gene flow between *R. cingulata* and *R. indifferens* if the two taxa come in contact. Microsatellite studies of *R. cingulata* [24] and *R. indifferens* [25,26] have also found little evidence for host-related differentiation between populations of these species attacking sweet or tart cherries versus their respective native cherry trees. Additionally, studies of non-host related RI reported only modest levels of premating isolation between *R. cingulata* and *R. indifferens* (isolation index = 0.27; [27]). Thus, all indications are that *R. cingulata* and *R. indifferens* are likely diverging gradually through an allopatric and non-ecological mode of speciation, but studies are needed to quantify the degree to which they have evolved intrinsic postzygotic isolation.

A recent microsatellite survey of *R. cingulata* and *R. indifferens* has implied, however, that the biogeography of these taxa may reflect a history of parapatry or clinal variation rather than a strict bifurcating mode of allopatric speciation [36]. In this regard, the microsatellites show that the two cherry-infesting flies form a clinal arc of populations across North America, and are genetically grading into one another through the Southwestern USA and Mexico [36]. *Rhagoletis indifferens* in the Pacific Northwest (PNW) and *R. cingulata* in the Eastern USA are diagnostically diverged from each other for a subset of microsatellite alleles [36]. In contrast, *R. cingulata* across the Southwestern USA and Northern Mexico possess mixtures of microsatellite allele frequencies grading from alleles characteristic of *R. indifferens* in the PNW to *R. cingulata* in the Eastern USA, as well as a subset of unique variants [36]. The implication is that *R. cingulata* and *R. indifferens* were once contiguous across the Southwestern USA and Northern Mexico during the last Wisconsin glaciation. However, it appears that *R. indifferens* became isolated in the PNW and *R. cingulata* fragmented across the Southwestern USA and Northern Mexico in the Holocene when warming in the last 9000 years increased the aridity of the region and forced previously low-lying woodlands and forests to move upslope on mountains, reducing and isolating populations [37,38,39,40]. Estimates of divergence times based on microsatellite data date the fragmentation of Southwestern USA and Northern Mexico cherry-infesting fly populations from those in the PNW and Eastern USA to ~5500 to 8400 years ago (ya) [36], coinciding well with the climatic warming in the Holocene.

The *R. cingulata* group, therefore, represents a system for discriminating ecological versus non-ecological drivers for adaptive radiation. *Rhagoletis cingulata* and *R. indifferens*, both cherry-infesting, provide a spatial and temporal framework for characterizing patterns of genetic differentiation, rates of population divergence, and the evolution of intrinsic RI, based on non-ecological processes. Complementary ecologically based patterns can be examined among sympatric black cherry- (*R. cingulata*)*,* olive- (*R. chionanthi* and *R. osmanthi*) and muttonwood-infesting (*R. turpiniae*) species, hypothesized to have ecologically speciated via host shifting. Specifically, we can compare rates of evolution of pre- and intrinsic postzygotic RI per unit time, inferred by the degree of genetic divergence, between sympatric species on different hosts and allopatric cherry-infesting species. From such comparisons, inferences can be made about the importance of ecological speciation versus non-ecological speciation and character displacement in initiating population divergence and fostering adaptive differentiation for these flies.

Here, we examine the roles that ecology, geography, and non-adaptive processes may have played in divergence among flies in the *R. cingulata* species group. To do so, we combine population genetic analyses of microsatellite and mitochondrial DNA (mtDNA) variation among all members of the group with tests of intrinsic postzygotic RI between two geographically isolated cherry-infesting species, *R. cingulata* and *R. indifferens*. The ecological theory predicts that the degree and rate evolution of host-related RI should be greater among sympatric *R. osmanthi*, *R. chionanthi*, and *R. cingulata* in the Southeastern USA and *R. turpiniae* and *R. cingulata* in Mexico, compared to allopatric *R. cingulata* and *R. indifferens* (Figure 1). Such a finding would support a primary role for divergent natural selection associated with sympatric host shifting in adaptive radiation within the group. If this is not the case, then non-adaptive speciation and character displacement following secondary contact may play a larger role in the radiation of these flies.

## 2. Materials and Methods 

### 2.1. Study Sites and Collections

Fruits infested with larvae of *R. cingulata* species group flies were collected from 2004–2012 at sites across North America (Figure 1, Table 1). Microsatellite and mtDNA data for *R. indifferens* and *R. cingulata* were taken from Saint Jean et al. [26] and Doellman et al. [36]. New microsatellite and mtDNA sequencing data in the current study were generated from three non-cherry-infesting populations of *R. cingulata* group flies: *R. chionanthi* (site 18, collected from fringe-tree at Perry, Georgia), *R. osmanthi* (site 19, collected from tea-olive at Lake Lizzie, Florida), and *R. turpiniae* (site 20, collected from muttonwood at Xalapa, Veracruz, Mexico). Flies were reared to adulthood in the laboratory using standard *Rhagoletis* husbandry methods [41], as described in Feder et al. [42] and Tadeo et al. [43]. Specimens were frozen and stored at −80 °C prior to genetic analysis.

### 2.2. Microsatellite Genotyping

DNA was isolated and purified from adult head or whole-body fly tissue from 420 flies from 18 sites for microsatellite analysis (Table 1) using PUREGENE extraction kits (Gentra Systems, Minneapolis, MN). Purified DNA samples were transferred to 96-well plates for microsatellite PCR amplification and genotyping. Flies were genotyped for 21 dinucleotide repeat microsatellite loci. Twelve of the loci (WCFF07, WCFF024, WCFF031, WCFF057, WCFF061B, WCFF067, WCFF083, WCFF084A, WCFF086A, WCFF093, WCFF0105, WCFF0111) were developed for *R. indifferens* by Maxwell et al. [44] and nine of the loci (P4, P27, P36, P37, P45, P50, P54, P71, P80) were developed originally for *R. pomonella* by Velez et al. [45], but also cross amplify and are polymorphic in cherry-infesting flies. Forward and reverse primers and the conditions used to PCR amplify microsatellites are described in Maxwell et al. [25,44] and Michel et al. [46], respectively. The 21 microsatellites were chosen because they displayed no systematic evidence for heterozygote deficiency from Hardy–Weinberg equilibrium due to null alleles, as determined using MICRO-CHECKER [47]. Genotyping was performed on a Beckman–Coulter CEQ8000, as described in Saint Jean et al. [26]. Microsatellite alleles were sized using the Fragment Analysis software provided by Beckman–Coulter (Brea, CA, USA). Size standards were included in each genotyping run. In addition, for a subset of runs, three to four *R. cingulata* from sites in the Eastern and Southwestern USA and Mexico, and *R. indifferens* from Woodland, WA (sites 2 and 3) were included to ensure that alleles were aligned and comparably scored.

### 2.3. Genetic Analysis of Microsatellites

An unrooted neighbor-joining genetic distance network for the 21 microsatellites based on Nei’s genetic distances between populations [48] was constructed using PowerMarker v3.25 [49]. Bootstrap support for each node was calculated based on 10,000 replicates across loci. Tests for evidence of genetic subdivision based on the microsatellites were performed using STRUCTURE v2.3.4 [50]. Ten replicate runs were conducted, under the admixture model with correlated allele frequencies and no prior population information, for *K* values ranging from 1–15 with a burn-in of 500,000 iterations and 1,000,000 data-collecting steps. Two methods were used to infer the number of clusters (subpopulations) that best fit the observed data [51]: the method of Evanno et al. [52] based on the rate of change in the natural logarithm (*Ln*) probability of data between successive *K* values (delta *K*), and Pritchard et al. [50] involving direct comparisons of the *Ln* probabilities of K. 

Divergence times, based on the microsatellites, were previously estimated in Doellman et al. [36] between cherry-infesting fly populations in the Eastern USA (site 15; South Bend, IN) and the PNW (site 4; Hood River, OR). Here, we expand on this analysis to include estimates of divergence time between 1) the Eastern USA (site 15) and Southern Mexico (site 9; San Martin, Texmelucan); 2) the PNW (site 4) and Southern Mexico (site 9); 3) *R. turpiniae* (site 20; Xalapa, Veracruz) and *R. cingulata* from Southern Mexico (site 10; Huamantla, Tlaxcala); and 4) nested divergence between *R. osmanthi* (site 19; Lake Lizzie, FL) and *R. chionanthi* (site 18; Perry, GA), and then, their common ancestor *R. cingulata* from the Southeastern USA (site 13; Live Oak, FL). 

Populations used for divergence estimates were selected from each region because they displayed the smallest pairwise Nei’s genetic divergence and/or were most geographically proximate, which made our estimates conservative. Divergence times were derived using the Metropolis-coupled Markov Chain Monte Carlo (MCMC) sampling algorithm in the program IMa2p [53,54,55]. Posterior probability distributions for divergence times were generated using non-informative priors for ancestral population size (θ), migration rates between populations (*m*_1_ and *m*_2_), and time since divergence (*t*) in generations, which for *R. cingulata* group flies, equals the number of years since separation, as these flies have one generation per year. To obtain an upper bound estimate for divergence time, *m*_1_ and *m*_2_ were both set to 0 under the assumption of no gene flow following the separation of *R. cingulata* group species. We implemented the step-wise mutation model for microsatellite evolution across a range of common mutation rates, 1 × 10^−4^, 1 × 10^−5^, and 6.3 × 10^−6^ per meiosis [56] but present results only for the latter here, as it is congruent with estimates for other insects, including *Drosophila* and pea aphids [57,58,59]. The 21 microsatellites genotyped conformed well to a dinucleotide repeat step-wise mutation model, with the exception that certain loci contained one or a few rare alleles differing from a strict 2 bp repeat pattern by a single nucleotide (see Table S1; [60]). In these cases, we increased or decreased the length of the allele by 1 bp. For each mutation rate and population pair analyzed, 10 MCMC simulations were run for at least 1 × 10^7^ iterations, with a thinning period of 10,000, after an initial burn-in of 3 × 10^7^ iterations, using 200 chains and heating parameters ha = 0.99 and hb = 0.4. Results are reported for the 10 pooled runs, which all converged to similar posterior distributions.

### 2.4. Mitochondrial DNA

Mitochondrial DNA sequence data were generated for a 609 bp fragment of the cytochrome oxidase II (COII) gene using the primers C2-J-3138 and TK-N-3782 [61]. Methods used for Sanger sequencing of mtDNA by the University of Arizona Genetics Core facility in Tucson, AZ can be found in Doellman et al. [36]. GenBank accession numbers for *R. cingulata* and *R. indifferens* mtDNA sequences from Doellman et al. [36] are KT221476-KT22179. In addition, new mtDNA sequence data were generated for *R. chionanthi* from site 18 (n = 5), *R. osmanthi* from site 19 (n = 1), and *R. turpiniae* from site 20 (n = 34; GenBank accession numbers MN366239-MN366243). A maximum parsimony mtDNA gene tree was constructed for COII haplotypes using PAUP*b8 (Swofford 2000). Two sequences from the outgroup species *R. suavis* from East Lansing, MI were used to root the haplotype network (GenBank accessions HQ677149, AY152493).

### 2.5. Crosses Testing for Postzygotic RI

Crosses testing for intrinsic postzygotic RI were performed as described in Tadeo et al. [43]. *Rhagoletis cingulata* collected as larvae infesting black cherry fruit from South Bend, IN and *R. indifferens* infesting sweet cherries in Vancouver, WA in 2015 were transported to the University of Notre Dame, Notre Dame, IN, where they were reared to adulthood following a five-month overwintering period as pupae. Adult flies were sexed within three days of eclosing, as *R. cingulata* and *R. indifferens* typically reach sexual maturity in 6–10 days [62]. Flies of the same sex were transferred to separate plexiglass holding cages (30 × 30 × 30 cm) in an incubation room (22 °C, 65% relative humidity, 14:10 L:D), with access to water and food (a 1:3 mixture of hydrolyzed protein and sugar). After one week, three male and three female sexually mature virgin flies were placed together in new cages with three 1.5 cm diameter agar spheres. The spheres were made by mixing 14.6 g of agar into 400 mL of hot water and 1.5 mL of red food coloring, and the hot mixture poured into 1.5 cm spherical molds. After cooling and prior to being hung in the mating cages, the spheres were wrapped in Parafilm. Dead flies were removed from the cages and substituted with new flies of the appropriate sex on a daily basis. Adult fly life span has been estimated from one to two months in the laboratory [43,62].

Reproductive isolation was assessed by the numbers of eggs laid and percentages of eggs hatched per mating. After four days, the agar spheres were removed from the cages, held in the incubation room, and replaced with freshly prepared spheres to allow for continued egg laying. Eggs that had been laid under the surface of the spheres were counted by inspecting the spheres under a light microscope and the position of eggs on the sphere noted by a pen mark. From days 2–7, eggs were monitored under the microscope once daily to see if they had hatched and larvae had tunneled into the sphere. In total, we set up four different non-choice mating treatments: two representing parental crosses of *R. indifferens* ♀ x *R. indifferens* ♂ (n = 21 total mating cages) and *R. cingulata* ♀ x *R. cingulata* ♂ (n = 6 cages), and two representing reciprocal hybrid crosses of *R. indifferens* ♀ x *R. cingulata* ♂ (n = 10 cages) and *R. cingulata* ♀ x *R. indifferens* ♂ (n = 14 cages). Tests for statistically significant differences in fertility and fecundity among cross types, as measured by the numbers of eggs laid, the percentages of eggs hatched, and the numbers of offspring produced (eggs laid x % hatched) were determined by ANOVA, considering each mating cage as an independent replicate. Tukey HSD post hoc comparisons were conducted following a significant ANOVA.

## 3. Results

### 3.1. Nuclear Encoded Microsatellites

**Figure 2:**
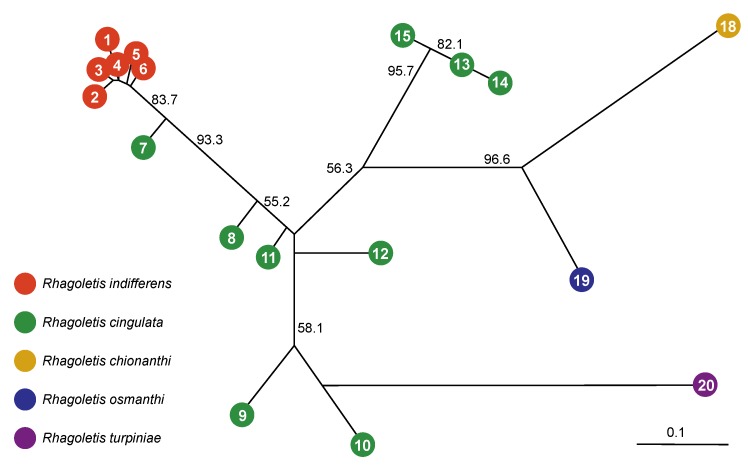
Neighbor-joining genetic distance network for *R. cingulata* group flies across North America based on 21 microsatellite loci. Also shown are bootstrap support levels for nodes based on 10,000 replicates. A Nei’s genetic distance of 0.1 is indicated by the scale bar.

The results for the neighbor-joining Nei’s genetic distance network based on microsatellites showed that host-associated divergence for *R. osmanthi*, *R. chionanthi*, and *R. turpiniae* was superimposed on a pattern of clinal geographic variation between the cherry-infesting species *R cingulata* and *R. indifferens* (Figure 2). The network implied that *R. turpiniae* is most closely genetically related to black cherry-infesting populations of *R. cingulata* in Southern Mexico, while the olive-attacking taxa *R. osmanthi* and *R. chionanthi* are sister taxa of intermediate genetic distance between black cherry-infesting forms of *R. cingulata* in Northern Mexico and the Eastern USA (Figure 2). Nei’s genetic distances between the three non-cherry-infesting taxa and local *R. cingulata* populations (range: 0.555–0.608) were comparable to those seen across the entirety of the range of cherry-infesting *R. cingulata* and *R. indifferens* in North America (range: 0.460–0.613; Figure 2; Table 2). 

In contrast, Nei’s genetic distances among cherry-infesting fly populations within the PNW, Southern Mexico, and Eastern USA regions were approximately an order of magnitude lower than that between the regions (range: 0.046–0.103; Table 2). The Nei’s genetic distance between the olive-attacking taxa *R. osmanthi* and *R. chionanthi* was also much higher than the differentiation within each geographic region of cherry-infesting populations (0.395; Table 2). Comparisons between populations attacking different hosts were not sympatric, although they were sampled from the same geographic region. Thus, the genetic distance estimates involving non-cherry-infesting fly populations could be inflated by geographic differentiation. However, geographic distances between cherry-infesting sites within regions were similar to or greater than those involving non-cherry-infesting flies, yet Nei’s genetic distances were still an order of magnitude lower for within-region comparisons of cherry-infesting flies. 

**Figure 3:**
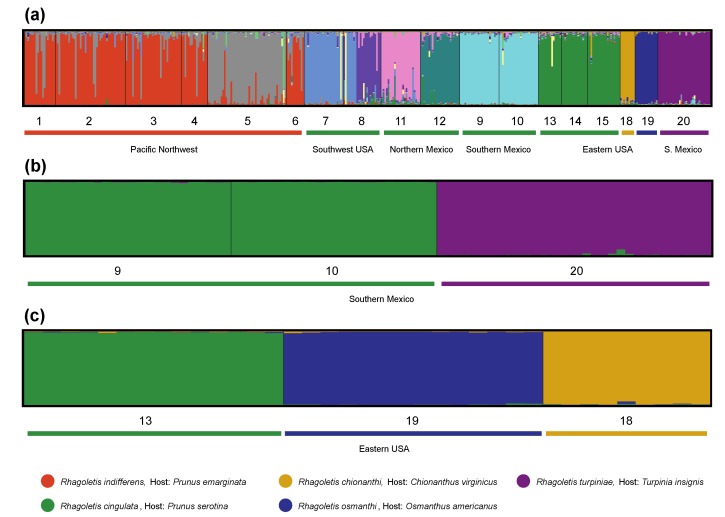
STRUCURE plots based on microsatellites for: (**a**) all *R. cingulata* group flies across North America with the best fit K = 13 subpopulations, as determined by the method of Pritchard et al. [50]; (**b**) Southern Mexican populations of *R. cingulata* (sites 9 and 10) and *R. turpiniae* (site 20) with the best fit K = 2, as determined by the method of Evanno et al. [52]; and (**c**) Southeastern USA populations of *R. cingulata* (site 13), *R. chionanthi*, (site 18), and *R. osmanthi* (site 19) with the best fit K = 3 [52].

Results from STRUCTURE analyses (Figure 3) were concordant with the characterization of host and geographic variation for *R. cingulata* group flies inferred from the genetic distance network (Figure 2). According to the method of Prichard et al. (2000), the number of subpopulations of *R. cingulata* group flies that best fit the data was K = 13 (Figure 3A; Table S2). *Rhagoletis indifferens* comprised two diverged demes, central WA (site 5) and the remainder of the PNW (sites 1–4 and 6). The six subpopulations within *R. cingulata* included Arizona, USA (site 7); Texas, USA (site 8); Los Lirios, Coahuila Northern Mexico (site 11); Monterrey, Nuevo Leon, Northern Mexico (site 12); both Southern Mexican populations (sites 9 and 10); and the Eastern USA (sites 13–15). *Rhagoletis turpiniae* (site 20), *R. osmanthi* (site 19) and *R. chionanthi* (site 18) each formed a distinct cluster. Three individuals were also identified from across all the populations that were assigned to two non-geographically clustered subpopulations. Based on the method of Evanno et al. (2005), the best fit number of subpopulations considering *R. turpiniae* versus the two *R. cingulata* populations in Southern Mexico was K = 2 (Figure 3B). One of the two subpopulations distinctly represented *R. turpiniae* with no individuals of mixed ancestry and the other subpopulation represented the two *R. cingulata* sites. Similarly, when considering geographically proximate *R. osmanthi*, *R. chionanthi*, and *R. cingulata* populations in the Southeastern USA, the best fit number of subpopulations was K = 3 (Figure 3C). The three subpopulations represented *R. osmanthi*, *R. chionanthi* and *R. cingulata*, respectively, each lacking individuals of immediate mixed ancestry.

Examination of the distribution of private microsatellite alleles for each taxon further substantiated the implications of the genetic distance network and STRUCTURE plots; *R. osmanthi*, *R. chionanthi*, and *R. turpiniae* represent host-associated lineages (species), distinct from the cherry-infesting *R. cingulata* and *R. indifferens* and each other (Table S1). For *R. turpiniae*, the microsatellites WCFF93 and P37 were fixed at a frequency of 1.0 for an allele not present in any other *R. cingulata* group fly (Table S1). The same was true for microsatellite P36 in *R. chionanthi*, while *R. osmanthi* had only three locus P37 alleles, none of which were genotyped in another *R. cingulata* group fly (Table S1). The pattern was more complicated concerning the two cherry-infesting flies *R. cingulata* and *R. indifferens*, as illustrated by the microsatellites WCFF57 and P37. Eastern USA populations of *R. cingulata* and PNW populations of *R. indifferens* possessed completely unique sets of alleles for these two loci. However, the alleles distinguishing Eastern USA *R. cingulata* and PNW *R. indifferens* were not autapomorphies in that they were also present: 1) in one or both of the native-olive infesting flies *R. osmanthi* and *R. chionanthi*; and 2) in *R. cingulata* populations in the Southwestern USA and Mexico. Indeed, for much of the nuclear genome, microsatellite allele frequency differences between PNW *R. indifferens* and Eastern USA *R. cingulata* graded into one another across the Southwestern USA and Mexico. Thus, the Southwestern USA and Northern Mexican populations of cherry-infesting flies genetically bridge the species gap between PNW *R. indifferens* and the Eastern USA *R. cingulata*, with respect to the nuclear genome. 

The estimated divergence time with the highest posterior probability between *R. osmanthi* and *R. chionanthi* in the Southeastern USA, assuming a microsatellite mutation rate of 6.3 × 10^−6^ per generation in the IMa2p analysis, was 8413 ya (95% credible interval (CI), 2881 to 20,794 ya). The separation of these two olive-infesting species from *R. cingulata* in the Eastern USA (site 13) was estimated to be 21,746 ya (95% CI 9365 to 56,032 ya). In Southern Mexico, the estimated divergence time between *R. turpiniae* and *R. cingulata* (site 10) was 15,079 ya (95% CI 7460 to 29,365 ya). In comparison, estimated divergence times for cherry-infesting flies at the ends of their distribution in the Eastern USA (site 15) versus PNW (site 4), Eastern USA (site 15) versus Southern Mexico (site 9), and PNW (site 4) versus Southern Mexico (site 9) were 15,079 ya (95% CI 7143 to 31,270), 23,651 ya (95% CI 14,732 to 50,371), and 14,444 ya (95% CI 6508 to 30,000), respectively (Table S3).

### 3.2. Mitochondrial DNA

**Figure 4:**
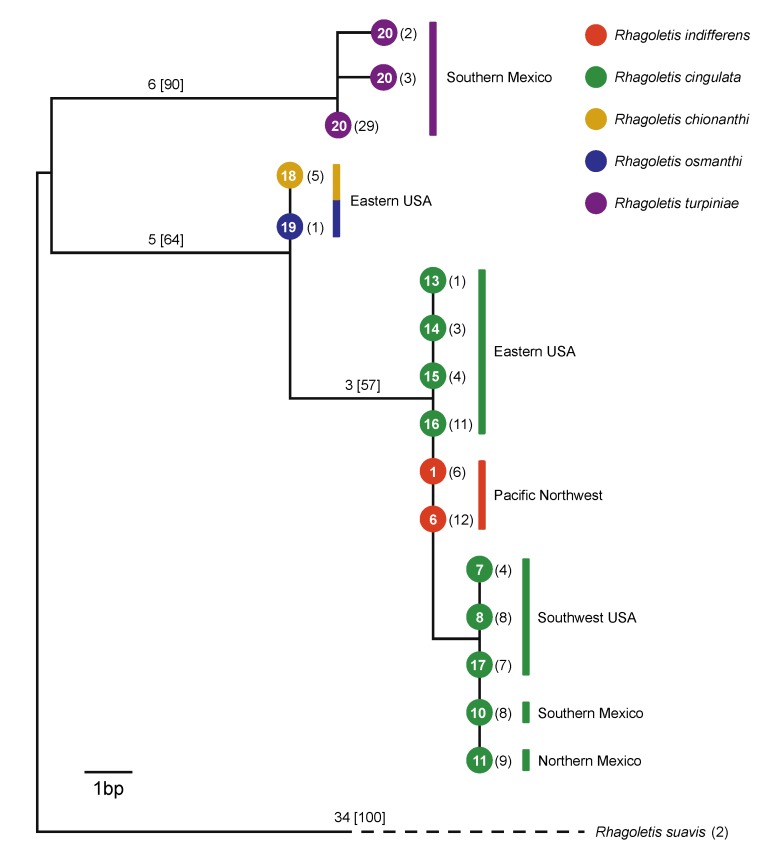
Mitochondrial DNA (mtDNA) maximum parsimony gene tree (51 steps) built from 609 total bp of cytochrome oxidase II (COII), sequenced for *R. cingulata* species group flies across North America. Values in parentheses indicate the number of identical haplotypes sequenced from different individuals within a population, with site number designated inside the nodes, as given in Table 1 and Figure 1. Also shown along the branches are the number of base pair substitutions, with the bootstrap support levels for nodes (based on 10,000 replicates) in brackets. The tree is rooted with the outgroup walnut husk-infesting fly, *R. suavis*, from East Lansing, Michigan.

The results for mtDNA showed both similarities to and differences from the nuclear-encoded microsatellite data (Figure 4). Similar to the microsatellites, different mtDNA COII haplotypes distinguished the muttonwood-infesting *R. turpiniae* and the two olive-infesting flies from *R. cingulata* and *R. indifferens* (Figure 4). However, unlike the microsatellites, mtDNA sequences did not differentiate *R. osmanthi* and *R. chionanthi*. The lack of mtDNA divergence between *R. osmanthi* and *R. chionanthi* was consistent, however, with these two populations representing related sister taxa, in concordance with the microsatellites (Figure 2). 

A second and more pronounced difference between the mtDNA results and the microsatellites, as previously observed by Doellman et al. [36], concerned the lack of mtDNA divergence displayed between Eastern USA populations of *R. cingulata* and PNW populations of *R. indifferens* (Figure 4). In contrast, these two flies showed significant microsatellite differentiation between populations in the Eastern USA versus PNW (Figure 2; Table 2). Moreover, while Southwestern USA and Mexican populations of *R. cingulata* appeared to bridge the nuclear genome differences between cherry-infesting flies in the Eastern USA and PNW, the same was not true for mtDNA. Indeed, the Southwestern USA and Mexican populations of *R. cingulata* possessed a derived COII haplotype that differed by a single base pair substitution from those of Eastern *R. cingulata* and PNW *R. indifferens* flies (Figure 4). Doellman et al. [36] observed an additional four base pair difference in the Southwestern and Mexican haplotype for a longer 1482 bp sequence of mtDNA that, in addition to COII, included the COI and t-RNA-Leu genes. Thus, populations of cherry-infesting flies in the Southwestern USA and Mexico could represent a distinct mtDNA matrilineage from the other *R. cingulata* group flies in North America.

### 3.3. Postzygotic RI

There was no evidence for significant intrinsic postzygotic RI between *R. cingulata* and *R. indifferens*. The mean number of eggs laid per mating cage did not differ significantly among the parental and reciprocal hybrid mating types (F_3, 47_ = 0.36, *P* = 0.78; Table 3). There was, however, a statistically significant difference in the percentages of egg hatch per cage among the four cross types (F_3, 47_ = 4.48, *P* < 0.008; Table 3). The highest percentage of egg hatch per cage was observed for the *R. indifferens* x *R. indifferens* parental cross (mean = 82.9% + 4.9% s.e., n = 21 cages). Crosses involving female *R. cingulata*, including the alternate *R. cingulata* x *R. cingulata* parental cross, displayed a significantly lower level of egg hatch (Table 3). The result suggests that rather than reflecting postzygotic RI, the variation in egg hatch rate represents an inherent difference between *R. cingulata* and *R. indifferens* in early egg survivorship, possibly associated with the agar spheres or lab rearing conditions we used in the study. The total number of eggs hatched did not differ significantly among the four cross types (F_3, 47_ = 0.54, *P* = 0.66; Table 3), despite the reduced egg hatch rate of the parental *R. cingulata* x *R. cingulata* cross.

## 4. Discussion

In general, our results support the ecological theory for adaptive radiation in the *R. cingulata* group. In this regard, the nuclear-encoded microsatellites imply that host association significantly affects patterns of genetic divergence and, by inference, generates ecologically based RI and induces speciation in *R. cingulata* group flies (Figure 2 and Figure 3; Table 2). In previous studies, Maxwell et al. [25], Smith et al. [24] and Saint Jean et al. [26] found little evidence for genetic differentiation between native and domesticated cherry-infesting populations of *R. cingulata* or *R. indifferens*. However, here we have shown that genetically distinct forms of *R. cingulata* group flies exist on non-cherry host plants, including native olives and muttonwood, in areas of geographic overlap (sympatry) with the cherry-infesting species *R. cingulata*. Thus, ecological speciation via host plant shifting appears probable in the *R. cingulata* group, as has been inferred for other *Rhagoletis* species groups [13,15,46,63,64,65,66]. However, different cherry hosts, even with differing fruiting phenologies, may not exert strong enough divergent selection pressures to significantly reduce gene flow and induce host race formation for *R. cingulata* or *R. indifferens*.

### 4.1. Divergence Timing and Ecological Versus Geographic Isolation

The estimated ages of divergence events in which host shifts generated new taxa in the *R. cingulata* group ranged from 8413 to 21,746 ya (Table S3). These divergence times are on the same scale as estimates for geographic divergence spanning ~5500 to 8400 ya, for fragmentation of cherry-infesting fly populations by increasingly warm and arid conditions across the Southwestern USA and Northern Mexico during the Holocene [36], and 14,444 to 23,651 ya, for isolation of cherry-infesting populations at the geographic extremes of their ranges in North America. Thus, the lengths of time required for ecological and non-ecological RI to accrue for these taxa likely did not differ substantially (Table 2 and Table S3). Our estimates of divergence times are conservative, based on a mutation rate of 6.3 × 10^−6^, which corresponds to the rate for *Drosophila* and pea aphids [57,58,59] and represents the low end of reported values in insects [56]. Nevertheless, using a higher mutation rate (e.g., 10^−4^) would not change the inference that host shifting in the *R. cingulata* group is occurring on a similar timeframe as the geographic separation of the taxa *R. cingulata* and *R. indifferens* in North America. It would only result in the history of these events being proportionately more recent (i.e., occurring hundreds rather than thousands of years ago), which seems less likely given the timing of major climatic changes in North America [37,39]. It is possible, however, that while we found no evidence for current gene flow among *R. osmanthi*, *R. chionanthi*, *R. turpiniae, and R. cingulata*, we may have underestimated their divergence times due to genetic exchange occurring during earlier formative stages of the speciation process, slowing their rate of genetic differentiation. 

We also note that we can only infer but not verify complete RI among extant populations of *R. osmanthi*, *R. chionanthi*, *R. turpiniae,* and *R. cingulata*, evolved primarily by divergent ecological selection. In this regard, we genetically analyzed one population each of *R. turpiniae*, *R. osmanthi*, and *R. chionanthi*, which were not sympatric (within 1 km) with sampled populations attacking alternate hosts. Considering our modest sample sizes (n = 9–32), it is possible that low-level gene flow is still occurring for these flies (Figure 3a,b), which requires future testing of sympatric populations to refute or confirm. In addition, studies of non-host related pre- and postzygotic RI involving mating trials and crosses among local populations of *R. turpiniae*, *R. osmanthi*, *R. chionanthi*, and *R. cingulata* are needed to discount a contributing role for other forms of non-ecologically based RI in the divergence of these taxa. However, the current overlap of cherry and non-cherry host plants and flies in the Southeastern USA and Southern Mexico and the estimated 8413 to 21,746 years of divergence among species in the *R. cingulata* group are consistent with the ecological hypothesis for adaptive radiation via sympatric host shifting for *R. turpiniae*, *R. osmanthi*, and *R. chionanthi* (Figure 1; Table S3).

### 4.2. Pre- and Postzygotic RI and Geographic Isolation

Our finding of no apparent intrinsic postzygotic RI between *R. indifferens* from the PNW and *R. cingulata* from the Eastern USA further implies that speciation in the *R. cingulata* group may proceed more slowly by non-ecological processes in allopatry than by adaptive divergence associated with sympatric host shifts. Our measures of inherent postzygotic RI were number of eggs laid and percentage of eggs hatched (Table 3). It is, therefore, possible that strong intrinsic postzygotic RI is manifested in F1 hybrids at later larval, pupal, or adult stages of the life cycle, or in later generation hybrids of mixed ancestry. However, greatly reduced rates of egg laying and percentages of eggs hatched, indicative of postzygotic RI, have been observed in interspecific crosses within other *Rhagoletis* species groups [23], and even between certain populations of *R. cingulata* [43].

Hood et al. [27] reported prezygotic isolation between *R. indifferens* from the PNW and *R. cingulata* from the Eastern USA; however, the level of prezygotic sexual isolation was modest (isolation index = 0.27). The two cherry-infesting flies do vary morphologically. Larvae of the two species differ in the number of papillae on the thoracic spiracles, with no overlap [67]. In addition, in the Eastern USA, ~80% of 350 *R. cingulata* examined from site 15 had an apical wing spot separated from the apical band by a hyaline area [36]. This wing spot was found at intermediate frequencies in the Southwestern USA (~61%; n = 562; sites 7 and 8) and is rare in *R. indifferens* in the PNW (~14%; n = 890; sites 4 and 5; [36]). Thus, there is evidence for some morphological and genetic divergence correlated with modest prezygotic isolation between *R. cingulata* and *R. indifferens*. However, cherry-infesting flies display a clinal pattern of geographic variation for both the apical wing spot and microsatellites across the Southwestern USA and Mexico, suggesting the recent fragmentation of a formerly clinally varying species rather than the strict bifurcation of a population into allopatrically isolated species [36]. Indeed, it is not clear whether *R. cingulata* and *R. indifferens* are sufficiently diverged and reproductively isolated such that they would remain distinct upon secondary contact. Thus, there is some uncertainty as to their currently recognized status as described species [18]. As a result, there may be little potential for character displacement to rapidly drive the ecological diversification of *R. cingulata* group flies, at least with respect to cherry-infesting populations.

### 4.3. Cytoplasmic Incompatibility and Patterns of mtDNA Variation

One additional consideration to note is that the geographic pattern of mtDNA haplotype variation suggests that cytoplasmic RI may exist in *R. cingulata*, potentially complicating the adaptive radiation story. In contrast to the clinal patterns displayed by microsatellites in cherry-infesting fly populations across North America, mtDNA shows a disjunct pattern. Specifically, *R. indifferens* in the PNW and *R. cingulata* in the Eastern USA share the same mtDNA haplotype, while *R. cingulata* from the Southwestern USA and Mexico possess a different haplotype (Figure 4). Doellman et al. [36] estimated the coalescence time for the two mtDNA haplotypes at 100,000–150,000 ya, which would predate the hypothesized fragmentation of cherry-infesting flies during the Holocene ~5500 to 8400 ya, as well as the origin of the non-cherry-infesting taxa attacking native olives and muttonwood in the Southeastern USA and Southern Mexico ~8400 to 21,800 ya. 

The geographic pattern of variation observed for mtDNA could potentially be explained by differential linage sorting or by the selective sweep of a favorable mtDNA variant (e.g., see [68,69,70]). However, Tadeo et al. [43] reported that hybrid crosses between *R. cingulata* from Kearneysville, West Virginia in the Eastern USA and from Coahuila in Northern Mexico (site 9) displayed a dramatic (over 95%) reduction in the numbers of eggs laid and hatched (near complete postzygotic RI) compared to parental crosses. Crosses between flies from Coahuila and Southern Mexico (Huamantla, site 11) showed a ~30% reduction in fertility, mainly those involving Coahuila males and Huamantla females [43]. In addition, there was a reduction in fertility up to 50% in “hybrid” compared to parental crosses in both directions between cherry-infesting flies from Huamantla and Kearneysville, but again not nearly as extreme as that seen between Northern Mexico and Eastern USA matings [43]. 

One possible explanation for these results is the presence of the endosymbiont *Wolbachia*. Schuler et al. [71] have reported the presence of the endosymbiont in *R. cingulata*, within both native North American and introduced European populations. The endosymbiont may have infected Southwestern USA and Mexican populations of *R. cingulata* and spread, as a consequence of its causing cytoplasmic incompatibility (CI), as has been shown for other insects including *Drosophila* flies [72], *Panonychus* mites [73], and *Nasonia* wasps [74,75,76,77,78]. If true, then the variable pattern of postzygotic isolation observed by Tadeo et al. [43] may be accounted for by the presence or absence of different strains of *Wolbachia* in the Eastern USA versus Mexican populations of *R. cingulata*. The observed pattern of mtDNA divergence would then reflect cytoplasmic hitchhiking with the endosymbiont. In addition, variation in mtDNA haplotypes and the occurrence of CI among races of the European cherry fruit fly, *R. cerasi* (L.), have been linked to different strains of *Wolbachia* [79,80]. Thus, rapid sweeps of *Wolbachia* and associated postzygotic RI have a precedent among *Rhagoletis* populations [71,81].

If *Wolbachia* is shown to be the cause for postzygotic RI between the Eastern USA and Mexican populations of *R. cingulata*, then the endosymbiont-related CI could be evolving faster between certain allopatric populations of cherry-infesting flies than intrinsic postzygotic RI (i.e., Batson–Dobzhansky–Muller incompatibilities). If true, then for endosymbionts to contribute to adaptive radiation, it must also be demonstrated that allopatric populations of cherry-infesting flies display bidirectional CI, and that different strains of bacteria responsible for bidirectional CI are not lost or exchanged between taxa following secondary contact. Moreover, it must be shown that the CI caused by *Wolbachia* facilitates character displacement. Thus, while there are reasons to speculate that *Wolbachia* could cause CI in the *R. cingulata* group and contribute to allopatric speciation, we have no evidence for endosymbionts being involved in adaptive radiation. In this regard, the current geographic distribution of cherry-infesting flies implies that if *Wolbachia*-caused CI were to contribute to ecological character displacement, then the process would likely occur at a slower rate and, thus, be of relatively less importance than ecological speciation for the adaptive radiation of *R. cingulata* group flies.

## 5. Conclusions

In conclusion, adaptive radiation may be common in nature, accounting for a significant portion of the diversity of life [1]. Through the study of actively diverging taxa, generalities may emerge regarding the processes responsible for adaptive radiation. One common theme is that adaptive radiation requires open ecological and/or geographic opportunity, involving resources or areas not utilized or occupied by competitors, predators or parasites [1]. Beyond this, however, it is not clear whether adaptive radiation is most often initiated by ecological speciation or non-ecological speciation followed by character displacement [1,6]. Here, we present evidence suggesting that divergent ecological selection associated with sympatric host plant shifting is a primary factor facilitating the adaptive radiation of fruit flies in the *R. cingulata* sibling species group. It is possible that host shifts occur to mitigate interspecific competition when fly species sharing a common host come into secondary contact. However, the biogeographic history and rates at which non-host related RI evolve in the *R. cingulata* group argue against a scenario of allopatry, secondary contact, and character displacement. Nevertheless, future studies involving crosses between *R. cingulata* and other sympatric, non-cherry-infesting species are needed to confirm that divergent host adaptation is the principal barrier to gene flow between these flies and that non-host related prezygotic and postzygotic isolation is lacking or weak. Whether endosymbiont-caused CI plays a prominent role in adaptive radiation also remains to be determined, however, and there are reasons to suspect that it operates at a slower pace than ecological divergence, at least in the *R. cingulata* group. Finally, it remains to be seen if the majority of groups radiate in a manner similar to *R. cingulata* flies. Phytophagous insects are the most diverse group of multicellular species on Earth and most are specialists [82]. Thus, it seems plausible that ecological speciation may contribute to the adaptive radiation of many phytophagous insects, as they take advantage of new host plant resource opportunities.

## Figures and Tables

**Table 1:** Site number designations, locations, latitude and longitude, and number of flies genotyped from sites for microsatellites (n). Sites 1–6 were collected from *Prunus emarginata* (bitter cherry), sites 7–17 from *P. serotina* (black cherry), site 18 from *Chionanthus virginicus* (fringe-tree), site 19 from *Osmanthus americanus* (tea-olive), and site 20 from *Turpinia insignis* (muttonwood).

Site	Site Location	Lat. N, Long. W.	n
1	Salmon Arm, British Columbia, Canada	50°54′54″, 119°21′26″	19
2	L. Kalama Road, Woodland, Washington	45°56′24″, 122°40′41″	43
3	Lewis River, Woodland, Washington	45°56′20″, 122°38′5″	34
4	Trout Creek Rd, Hood River, Oregon	45°32′10″, 121°37′14″	16
5	Ronald, Kittitas County, Washington	47°23′50″, 121°02′67″	48
6	Somers, Montana	48°05′24″, 114°13′48″	11
7	Chiricahua Mountains, Arizona	31°55′44″, 109°5′15″	24
8	Davis Mountains, Texas	30°38′47″, 104°01′08″	23
9	San Martin, Texmelucan, Mexico	19°16′ 20″, 98°25′03″	24
10	Huamantla, Tlaxcala, Mexico	19°18′54″, 97°52′28″	24
11	Los Lirios, Coahuila, Mexico	25°20′58″, 100°17′46″	24
12	Nueva Leon, Mexico	25°48′48″, 100°22′02″	24
13	Live Oak, Suwannee County, Florida	30°22′06″, 83°14′38″	14
14	Green Creek, Cape May, New Jersey	39°02′11″, 74°54′05″	16
15	South Bend, Indiana	41°45′28″, 86°12′4″	20
16	Urbana, Illinois	45°38′25″, 122°35′33″	mtDNA
17	Gila Cliff Dwelling Mon., New Mexico	33°10′44″, 108°12′14″	mtDNA
18	Perry, Georgia	32°27′19″, 83°43′41″	9
19	Lake Lizzie, Florida	27°11′4″, 80°50′26″	15
20	Xalapa, Veracruz, Mexico	19°32′39″, 96°57′04″	32

**Table 2:** Mean overall Nei’s genetic distances (± s.e., where appropriate) between *R. cingulata* species group populations involving comparisons at varying levels of biological organization and geographic structure across North America. n = number of pairwise genetic distance estimates in the given comparison.

Level of Comparison	Region	n	Distance (s.e.)
Between species that co-occur in region			
*R. chionanthi* versus *R. cingulata*	Southeastern USA	1	0.608
*R. osmanthi* versus *R. cingulata*	Southeastern USA	1	0.555
*R. osmanthi* versus *R. chionanthi*	Southeastern USA	1	0.395
*R. turpiniae* versus *R. cingulata*	Southern Mexico	2	0.574 (0.086)
Between species in different geographic regions			
*R. indifferens* versus *R. cingulata*	Pacific Northwest (PNW)/Eastern USA	18	0.613 (0.014)
*R. indifferens* versus *R. cingulata*	PNW/Southern Mexico	12	0.463 (0.007)
Within species between different geographic regions			
*R. cingulata*	Eastern USA/Southern Mexico	6	0.460 (0.033)
Within species in same geographic region			
*R. indifferens*	PNW	10	0.046 (0.004)
*R. cingulata*	Eastern USA	3	0.060 (0.012)
*R. cingulata*	Southern Mexico	1	0.103

**Table 3:** Mean number of eggs laid per cage, mean percentage of eggs hatched per cage, and mean number of eggs hatched per cage for parental and reciprocal crosses between *R. cingulata* from the Eastern USA and *R. indifferens* from the Pacific Northwest. Letters denote significantly different means, according to Tukey HSD.

Cross (m x f)	n Cages	Mean Eggs Laid ± s.e.	Percent Egg Hatch ± s.e.	Mean n Eggs Hatched ± s.e.
*R. indifferens* x *R. indifferens*	21	29.2 ± 5.5	83.0 ± 4.9^a^	25.0 ± 5.0
*R. cingulata* x *R. indifferens*	10	41.5 ± 6.8	52.0 ± 11.5^ab^	20.8 ± 7.1
*R. indifferens* x *R. cingulata*	14	35.7 ± 12.3	64.0 ± 6.5^b^	22.8 ± 7.6
*R. cingulata* x *R. cingulata*	6	34.7 ± 9.7	46.0 ± 14.5^b^	11.2 ± 4.5
F_3,47_		0.36	4.48	0.541
*p*		0.782	0.0076	0.656

## Supplementary Materials

The following are available online at https://www.mdpi.com/2075-4450/10/9/275/s1, Figure S1: Range of *T. insignis* in Veracruz, Mexico, Table S1: Population level microsatellite allele frequencies, Table S2: Statistics from STRUCUTRE analyses, Table S3: Divergence time estimates from IMa2p analyses.
