# Supplementary material for: Geographic and Ecological Dimensions of Host Plant-Associated Genetic Differentiation and Speciation in the Rhagoletis cingulata (Diptera: Tephritidae) Sibling Species Group
Source: Insects. 2019 Aug 29;10(9):275. doi: 10.3390/insects10090275 (PMC6780410; doi:10.3390/insects10090275)
Supplement: Supplementary file 1 [file insects-10-00275-s001.zip › RcingGroupSI.docx]

**Supplemental Information**

**Figure S1.** The documented range of *Turpinia insignis* in Veracruz, Mexico, the site of the only known *R. turpiniae* infestations (Sosa 1998; Hernandez Ortiz 1993; Aluja et al. 2001). Also indicated are the collection sites for *R. cingulata* (sites 9 and 10) and *R. turpiniae* (site 20).

**Table S1.** See attached spreadsheet for population-level microsatellite allele frequency data.

**Table S2.** Mean estimated *Ln* likelihood, standard deviation, and Pr(K=k) (Pritchard et al. 2000) or ∆K (Evanno et al., 2005) calculated across ten replicate Structure analyses, using 500,000 burn-in iterations followed by 1,000,000 MCMC repetitions under the correlated allele frequencies with admixture model. A) All 15 cherry-infesting fly populations (*R. cingulata* and *R. indifferens*) and one population each of *R. turpiniae*, *R. osmanthi*, and *R. chionanthi*, for K = 1–15. The grey row indicates the best fit number of subpopulations for K = 13. B) Southern Mexican populations of *R. cingulata* (sites 9 and 10) and *R. turpiniae* (site 20), with the best fit number of subpopulations of K = 2. C) Southeastern USA populations of *R. cingulata* (site 13), *R. chionanthi*, (site 18), and *R. osmanthi* (site 19) with the best fit number of subpopulations of K = 3.

A)

| *K* | *Ln Lik* | *σ* | *Pr(K=k)* |
| --- | --- | --- | --- |
| 1 | -25424.50 | 0.09 | 0 |
| 2 | -21570.20 | 0.42 | 0 |
| 3 | -20199.04 | 180.99 | 0 |
| 4 | -19425.41 | 4.25 | 0 |
| 5 | -18915.47 | 234.67 | 0 |
| 6 | -18569.98 | 304.68 | 0 |
| 7 | -24250.94 | 15743.22 | 0 |
| 8 | -19233.39 | 2874.90 | 0 |
| 9 | -17553.78 | 126.87 | <<0.001 |
| 10 | -18732.60 | 4038.31 | <<0.001 |
| 11 | -17342.16 | 130.99 | <<0.001 |
| 12 | -17438.92 | 594.66 | <<0.001 |
| 13 | -17212.94 | 78.22 | 0.99 |
| 14 | -17450.63 | 851.56 | <<0.001 |
| 15 | -17385.67 | 280.32 | <<0.001 |

B)

| *K* | *Ln Lik* | *σ* | Δ*K* |
| --- | --- | --- | --- |
| 1 | -4611.04 | 0.34 | - |
| 2 | -3844.69 | 0.14 | 4659.25 |
| 3 | -3753.53 | 1.21 | 31.52 |

C)

| *K* | *Ln Lik* | *σ* | Δ*K* |
| --- | --- | --- | --- |
| 1 | -2187.03 | 0.49 | - |
| 2 | -1959.64 | 14.87 | 3.37 |
| 3 | -1782.36 | 0.13 | 1824.58 |

**Table S3.** Microsatellite based divergence times calculated in IMa2p, assuming a mutation rate of 6.3 × 10^-6^ per generation. Results are reported as the point estimate of the number of years since divergence (ya), along with the 95% credible interval (CI). A) Estimated divergence among species with differing host associations in the *R. cingulata* group. B) Estimated divergence among geographically isolated populations of cherry-infesting flies. See Table 1 and Figure 1 for detailed site information.

A)

| *Species 1* | *Site 1* | *Species 2* | *Site 2* | *Point Est. (ya)* | *95% CI* |
| --- | --- | --- | --- | --- | --- |
| *R. osmanthi* | 19 | *R. chionanthi* | 18 | 8,413 | 2,881 - 20,794 |
| *R. cingulata* | 13 | *R. chionanthi, R. osmanthi* | 18,19 | 21,746 | 9,365 - 56,032 |
| *R. cingulata* | 10 | *R. turpiniae* | 20 | 15,079 | 7,460 - 29,365 |

B)

| *Region 1* | *Site 1* | *Region 2* | *Site 2* | *Point Est. (ya)* | *95% CI* |
| --- | --- | --- | --- | --- | --- |
| Eastern USA | 15 | PNW | 4 | 15,079 | 7,143 - 31,270 |
| Eastern USA | 15 | Southern MX | 9 | 23,651 | 14,732 - 50,371 |
| PNW | 4 | Southern MX | 9 | 14,444 | 6,508 - 30,000 |
